# 3,3,6,6-Tetrakis­(hydroxy­meth­yl)-1,2,4,5-tetra­zinane tetra­hydrate

**DOI:** 10.1107/S1600536809045590

**Published:** 2009-11-04

**Authors:** S. Kongsutjarit, P. Thamyongkit, N. Muangsin, N. Chaichit, Amorn Petsom, Seik Weng Ng

**Affiliations:** a Department of Chemistry, Faculty of Science, Chulalongkorn University, Bangkok 10330, Thailand; b Department of Physics, Faculty of Science and Technology, Thammasart University, Pathum Thani 12121, Thailand; c Department of Chemistry, University of Malaya, 50603 Kuala Lumpur, Malaysia

## Abstract

In the title compound, C_6_H_16_N_4_O_4_·4H_2_O, the tetra­zinane mol­ecule lies across an inversion centre. The tetra­zinane ring adopts a chair conformation, and all imino H atoms occupy axial positions. In the crystal, adjacent mol­ecules are linked through O—H⋯O, O—H⋯N and N—H⋯O hydrogen bonds with water mol­ecules generating a three-dimensional network.

## Related literature

For the synthesis of hexa­hydro-1,2,4,5-tetra­zine derivatives by condensing aldehydes with hydrazine, see: Skorianetz & Kovats (1970 ▶). For the synthesis of the 3,6-dimethyl homolog, see: Sun *et al.* (2003 ▶); Zhou *et al.* (1999 ▶).
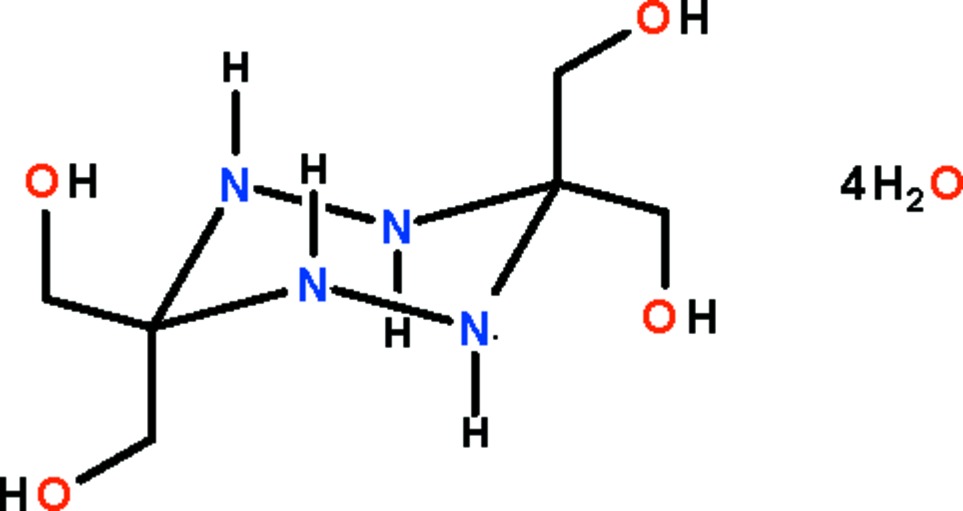


## Experimental

### 

#### Crystal data

C_6_H_16_N_4_O_4_·4H_2_O
*M* *_r_* = 280.29
Triclinic, 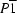

*a* = 6.3067 (1) Å
*b* = 7.0317 (2) Å
*c* = 8.4015 (2) Å
α = 71.010 (1)°
β = 74.424 (1)°
γ = 85.055 (1)°
*V* = 339.36 (1) Å^3^
*Z* = 1
Mo *K*α radiation
μ = 0.12 mm^−1^
*T* = 296 K
0.40 × 0.40 × 0.40 mm

#### Data collection

Bruker SMART APEXII diffractometer
Absorption correction: none
10198 measured reflections
4231 independent reflections
3630 reflections with *I* > 2σ(*I*)
*R*_int_ = 0.018

#### Refinement

*R*[*F* ^2^ > 2σ(*F* ^2^)] = 0.042
*wR*(*F* ^2^) = 0.137
*S* = 1.01
4231 reflections
114 parameters
8 restraints
H atoms treated by a mixture of independent and constrained refinement
Δρ_max_ = 0.93 e Å^−3^
Δρ_min_ = −0.63 e Å^−3^

### 

Data collection: *APEX2* (Bruker, 2005 ▶); cell refinement: *SAINT* (Bruker, 2005 ▶); data reduction: *SAINT*; program(s) used to solve structure: *SHELXS97* (Sheldrick, 2008 ▶); program(s) used to refine structure: *SHELXL97* (Sheldrick, 2008 ▶); molecular graphics: *X-SEED* (Barbour, 2001 ▶); software used to prepare material for publication: *publCIF* (Westrip, 2009 ▶).

## Supplementary Material

Crystal structure: contains datablocks global, I. DOI: 10.1107/S1600536809045590/ci2961sup1.cif

Structure factors: contains datablocks I. DOI: 10.1107/S1600536809045590/ci2961Isup2.hkl

Additional supplementary materials:  crystallographic information; 3D view; checkCIF report

## Figures and Tables

**Table 1:** Hydrogen-bond geometry (Å, °)

*D*—H⋯*A*	*D*—H	H⋯*A*	*D*⋯*A*	*D*—H⋯*A*
O1—H1*O*⋯O1*W*	0.85 (1)	1.87 (1)	2.704 (1)	166 (2)
O2—H2*O*⋯O2*W* ^i^	0.86 (1)	1.87 (1)	2.723 (1)	171 (2)
N1—H1*N*⋯O2^ii^	0.86 (1)	2.23 (1)	3.036 (1)	155 (1)
N2—H2*N*⋯O1*W* ^iii^	0.87 (1)	2.36 (1)	3.130 (1)	148 (1)
O1*W*—H1*W*1⋯O2*W* ^iv^	0.86 (1)	1.92 (1)	2.782 (1)	172 (2)
O1*W*—H1*W*2⋯N2^v^	0.86 (1)	2.03 (1)	2.869 (1)	166 (2)
O2*W*—H2*W*1⋯O1	0.84 (1)	1.92 (1)	2.759 (1)	175 (2)
O2*W*—H2*W*2⋯N1^vi^	0.84 (1)	2.02 (1)	2.853 (1)	171 (2)

Symmetry codes: (i) 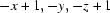; (ii) 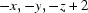; (iii) 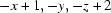; (iv) 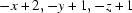; (v) 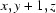; (vi) 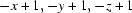.

**Fig. 1.**
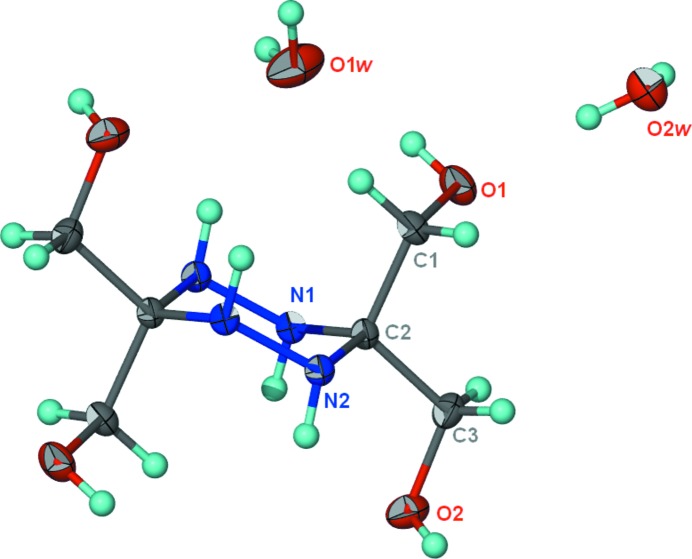
Displacement ellipsoid plot (Barbour, 2001) of C6H16N4O4.4H2O at the 50% probability level. H atoms are drawn as spheres of arbitrary radius. Unlabelled atoms in the tetrazinane derivative are related to labelled atoms by the symmetry operation (1-x, -y, 2-z). Two symmetry related water molecules are not shown.

## supplementary crystallographic information

### Experimental

Dihydroxyacetone (0.90 g, 10 mmol) and hydrazine hydrate (0.49 ml, 10 mmol) in ethanol (50 ml) were heated for 12 h. Slow evaporation of the solvent gave colourless crystals in 80% yield. The formulation of the organic molecule was established by ^1^H and ^13^C NMR as well as by mass spectroscopies.

### Refinement

The amino and water H-atoms were located in a difference Fourier map, and were refined with a distance restraint of N-H = O-H = 0.85 (1) Å; their U_iso_ parameters were freely refined. Carbon-bound H-atoms were placed in calculated positions (C-H = 0.97 Å) and were included in the refinement in the riding model approximation, with *U_iso_*(H) set to 1.2*U_eq_*(C). The highest peak and the deepest hole are located 0.73 and 0.58 Å from O1W. Although the displacement parameters of atom O1W are relatively large, no disorder is expected as its H-atoms could be located and refined.

### Crystal data

**Table:**

C_6_H_16_N_4_O_4_·4H_2_O	*Z* = 1
*M**_r_* = 280.29	*F*(000) = 152
Triclinic, *P*1	*D*_x_ = 1.371 Mg m^−^^3^
Hall symbol: -P 1	Mo *K*α radiation, λ = 0.71073 Å
*a* = 6.3067 (1) Å	Cell parameters from 6318 reflections
*b* = 7.0317 (2) Å	θ = 3.1–40.2°
*c* = 8.4015 (2) Å	µ = 0.12 mm^−^^1^
α = 71.010 (1)°	*T* = 296 K
β = 74.424 (1)°	Cube, colourless
γ = 85.055 (1)°	0.40 × 0.40 × 0.40 mm
*V* = 339.36 (1) Å^3^	

### Data collection

**Table:**

Bruker SMART APEXII diffractometer	3630 reflections with *I* > 2σ(*I*)
Radiation source: fine-focus sealed tube	*R*_int_ = 0.018
graphite	θ_max_ = 40.2°, θ_min_ = 3.1°
φ and ω scans	*h* = −11→11
10198 measured reflections	*k* = −12→12
4231 independent reflections	*l* = −15→15

### Refinement

**Table:**

Refinement on *F*^2^	Primary atom site location: structure-invariant direct methods
Least-squares matrix: full	Secondary atom site location: difference Fourier map
*R*[*F*^2^ > 2σ(*F*^2^)] = 0.042	Hydrogen site location: inferred from neighbouring sites
*wR*(*F*^2^) = 0.137	H atoms treated by a mixture of independent and constrained refinement
*S* = 1.01	*w* = 1/[σ^2^(*F*_o_^2^) + (0.0853*P*)^2^ + 0.0377*P*] where *P* = (*F*_o_^2^ + 2*F*_c_^2^)/3
4231 reflections	(Δ/σ)_max_ = 0.001
114 parameters	Δρ_max_ = 0.93 e Å^−^^3^
8 restraints	Δρ_min_ = −0.63 e Å^−^^3^

### Fractional atomic coordinates and isotropic or equivalent isotropic displacement parameters (Å^2^)

**Table:**

	*x*	*y*	*z*	*U*_iso_*/*U*_eq_	
O1	0.62982 (10)	0.32676 (8)	0.59353 (6)	0.03101 (11)	
O2	0.12442 (8)	−0.12559 (9)	0.85708 (8)	0.03186 (11)	
O1W	0.74166 (12)	0.53141 (12)	0.78141 (13)	0.0496 (2)	
O2W	0.81111 (10)	0.46492 (8)	0.23874 (7)	0.03194 (11)	
N1	0.35398 (7)	0.15628 (7)	0.93656 (6)	0.01863 (8)	
N2	0.53643 (7)	−0.16882 (6)	0.93673 (6)	0.01882 (8)	
C3	0.30946 (11)	−0.01190 (10)	0.73791 (8)	0.02651 (11)	
H3A	0.2599	0.1154	0.6697	0.032*	
H3B	0.3856	−0.0844	0.6586	0.032*	
C1	0.67667 (9)	0.13379 (9)	0.69956 (7)	0.02297 (10)	
H1A	0.7819	0.1472	0.7609	0.028*	
H1B	0.7435	0.0517	0.6262	0.028*	
C2	0.46871 (8)	0.02767 (7)	0.83271 (6)	0.01806 (9)	
H1O	0.661 (3)	0.409 (2)	0.640 (2)	0.051 (4)*	
H2O	0.140 (3)	−0.2396 (16)	0.839 (2)	0.052 (4)*	
H1W1	0.8827 (15)	0.535 (3)	0.765 (2)	0.057 (4)*	
H1W2	0.688 (3)	0.6353 (19)	0.810 (2)	0.055 (4)*	
H2W1	0.753 (2)	0.430 (2)	0.3464 (12)	0.053 (4)*	
H2W2	0.754 (3)	0.5766 (18)	0.198 (3)	0.069 (5)*	
H1N	0.2237 (14)	0.1097 (17)	0.9941 (14)	0.026 (2)*	
H2N	0.4192 (16)	−0.2420 (16)	0.9932 (15)	0.027 (2)*	

### Atomic displacement parameters (Å^2^)

**Table:**

	*U*^11^	*U*^22^	*U*^33^	*U*^12^	*U*^13^	*U*^23^
O1	0.0395 (3)	0.0239 (2)	0.02234 (19)	−0.00194 (17)	−0.00506 (17)	0.00069 (15)
O2	0.02304 (19)	0.0353 (2)	0.0423 (3)	−0.00251 (16)	−0.00474 (17)	−0.0211 (2)
O1W	0.0348 (3)	0.0488 (4)	0.0794 (6)	0.0026 (3)	−0.0098 (3)	−0.0437 (4)
O2W	0.0353 (2)	0.0254 (2)	0.0306 (2)	0.00639 (17)	−0.00462 (18)	−0.00747 (17)
N1	0.01946 (16)	0.01761 (16)	0.01842 (16)	0.00258 (12)	−0.00506 (12)	−0.00561 (12)
N2	0.02248 (17)	0.01557 (15)	0.01876 (16)	0.00090 (12)	−0.00525 (12)	−0.00611 (12)
C3	0.0297 (2)	0.0293 (3)	0.0242 (2)	−0.00180 (19)	−0.01135 (18)	−0.00917 (19)
C1	0.0244 (2)	0.0221 (2)	0.01861 (18)	−0.00032 (16)	−0.00180 (15)	−0.00427 (15)
C2	0.02086 (18)	0.01704 (17)	0.01609 (16)	0.00065 (13)	−0.00472 (13)	−0.00510 (13)

### Geometric parameters (Å, °)

**Table:**

O1—C1	1.4169 (7)	N1—H1N	0.86 (1)
O1—H1O	0.851 (9)	N2—N1^i^	1.4441 (6)
O2—C3	1.4198 (9)	N2—C2	1.4724 (6)
O2—H2O	0.86 (1)	N2—H2N	0.87 (1)
O1W—H1W1	0.86 (1)	C3—C2	1.5305 (8)
O1W—H1W2	0.86 (1)	C3—H3A	0.97
O2W—H2W1	0.84 (1)	C3—H3B	0.97
O2W—H2W2	0.84 (1)	C1—C2	1.5382 (7)
N1—N2^i^	1.4441 (6)	C1—H1A	0.97
N1—C2	1.4712 (7)	C1—H1B	0.97
			
C1—O1—H1O	105.1 (11)	C2—C3—H3B	109.4
C3—O2—H2O	104.1 (11)	H3A—C3—H3B	108.0
H1W1—O1W—H1W2	107.6 (16)	O1—C1—C2	112.12 (5)
H2W1—O2W—H2W2	105.0 (18)	O1—C1—H1A	109.2
N2^i^—N1—C2	113.59 (4)	C2—C1—H1A	109.2
N2^i^—N1—H1N	106.4 (8)	O1—C1—H1B	109.2
C2—N1—H1N	110.2 (8)	C2—C1—H1B	109.2
N1^i^—N2—C2	113.72 (4)	H1A—C1—H1B	107.9
N1^i^—N2—H2N	107.4 (8)	N1—C2—N2	114.01 (4)
C2—N2—H2N	108.2 (8)	N1—C2—C3	107.44 (4)
O2—C3—C2	111.33 (5)	N2—C2—C3	107.54 (4)
O2—C3—H3A	109.4	N1—C2—C1	110.36 (4)
C2—C3—H3A	109.4	N2—C2—C1	107.54 (4)
O2—C3—H3B	109.4	C3—C2—C1	109.89 (4)
			
N2^i^—N1—C2—N2	47.54 (6)	O2—C3—C2—N1	−65.11 (6)
N2^i^—N1—C2—C3	166.60 (4)	O2—C3—C2—N2	58.02 (6)
N2^i^—N1—C2—C1	−73.60 (5)	O2—C3—C2—C1	174.80 (5)
N1^i^—N2—C2—N1	−47.60 (6)	O1—C1—C2—N1	−54.32 (6)
N1^i^—N2—C2—C3	−166.60 (4)	O1—C1—C2—N2	−179.24 (4)
N1^i^—N2—C2—C1	75.09 (5)	O1—C1—C2—C3	63.98 (6)

Symmetry codes: (i) −*x*+1, −*y*, −*z*+2.

### Hydrogen-bond geometry (Å, °)

**Table:**

*D*—H···*A*	*D*—H	H···*A*	*D*···*A*	*D*—H···*A*
O1—H1O···O1W	0.85 (1)	1.87 (1)	2.704 (1)	166 (2)
O2—H2O···O2W^ii^	0.86 (1)	1.87 (1)	2.723 (1)	171 (2)
N1—H1N···O2^iii^	0.86 (1)	2.23 (1)	3.036 (1)	155 (1)
N2—H2N···O1W^i^	0.87 (1)	2.36 (1)	3.130 (1)	148 (1)
O1W—H1W1···O2W^iv^	0.86 (1)	1.92 (1)	2.782 (1)	172 (2)
O1W—H1W2···N2^v^	0.86 (1)	2.03 (1)	2.869 (1)	166 (2)
O2W—H2W1···O1	0.84 (1)	1.92 (1)	2.759 (1)	175 (2)
O2W—H2W2···N1^vi^	0.84 (1)	2.02 (1)	2.853 (1)	171 (2)

Symmetry codes: (ii) −*x*+1, −*y*, −*z*+1; (iii) −*x*, −*y*, −*z*+2; (i) −*x*+1, −*y*, −*z*+2; (iv) −*x*+2, −*y*+1, −*z*+1; (v) *x*, *y*+1, *z*; (vi) −*x*+1, −*y*+1, −*z*+1.
